# Quality of life results of balloon kyphoplasty versus non surgical management for osteoporotic vertebral fractures in Germany

**DOI:** 10.1186/2191-1991-1-7

**Published:** 2011-07-20

**Authors:** Daniela Eidt-Koch, Wolfgang Greiner

**Affiliations:** 1 Faculty of Public Health Services, Ostfalia - University of Applied Services, Wolfsburg, Germany; 2 Health Economics and Health Care Management, University of Bielefeld, Bielefeld, Germany

**Keywords:** osteoporosis, vertebral compression fracture, quality of life, balloon kyphoplasty, Germany

## Abstract

**Background:**

To compare improvement in quality of life (QoL) and symptoms' relief in vertebral compression fractures (VCF) due to osteoporosis for patients undergoing balloon kyphoplasty (BKP) to those undergoing non-surgical management (NSM) in a real-life setting.

**Methods:**

In this prospective, comparative study, quality-of-life was evaluated in eight centres in Germany between 2005 and 2008, for 82 patients, with the EQ-5D questionnaire, and the Roland Morris Disability Questionnaire (RMDQ).

**Results:**

BKP patients demonstrated a statistical and clinical significant higher improvement in EQ-5D than NSM patients, 0.44 and 0.25 from baseline to 12 months, respectively. Moreover, BKP patients showed a clinically relevant improvement in the RMDQ by 6.25 from baseline to 12 months, whereas NSM patients had no significant improvement in the RMDQ.

**Conclusions:**

This study demonstrates for VCF patients that in real-life quality of life for BKP patients improves more than for NSM patients; confirming the results of a large randomized clinical trial.

## Background

The social burden of osteoporosis has been increasing during the last decades worldwide due to aging of populations and potentially to changes in life style. The WHO has ranked osteoporosis among the ten most important diseases: "Osteoporosis has been recognized as an established and well-defined disease that affects more than 75 million people in the United States, Europe and Japan. It causes more than 8.9 million fractures annually worldwide, of which more than 4.5 million occur in the Americas and Europe" [1]. Osteoporosis is characterized by reduced bone mineral density which leads to destruction of bone microstructure. At early stage, there are no specific symptoms. In advanced stages, increased risk of bone fractures in the spine, hips or arms can be observed during daily routine activities [2-4]. Vertebral compression fractures (VCF) due to osteoporosis have been demonstrated to heavily impact patients' survival. In the US, Medicare patients with a VCF had an overall mortality rate twice higher than patients without it after adjusting for co-morbidities, with the greatest mortality rate in younger patients [5].

Two types of disease management are available to treat VCF. The usual treatment is *non-surgical management *(NSM). In this treatment option, analgesics can be used to relieve pain as well as bed rest, physiotherapy and/or back bracing in order to stabilize the vertebral fracture. NSM often leads to long hospital stays, slow pain decrease, and sometimes persistence of pain. Additionally, brace often limits patients' mobility, reduces strength in back muscles and doesn't revert kyphotic deformity. Therefore, even though NSM can decrease pain symptoms, it doesn't solve vertebral body height loss, kyphosis and reduced pulmonary function.

Other treatment options exist to better relieve pain symptoms and restore spine curvature. Among those treatments, *balloon kyphoplasty *(BKP) is a percutaneous vertebral augmentation procedure for the treatment of painful, acute vertebral fractures, designed to restore vertebral body height and shown to reduce kyphosis, in addition to relieve pain immediately. Due to height restoration and stabilization of the vertebral body an instantaneous pain relief can occur [6]. BKP has already been shown to improve quality-of-life, function, mobility and pain over NSM and more rapidly in VCF in a prospective randomized clinical trial over 12 months [7].

This analysis aims at comparing improvement in QoL in VCF patients due to BKP versus NSM in a real-life health care setting. Even though prospective controlled studies have been already published on BKP [8,9], this prospective multicentre study done in German real-life hospital settings is the first one collecting both QoL measures and treatment costs over 12 months. Moreover, the QoL measures used in this study are directly comparable with the ones used in the FREE randomized clinical trial of BKP [7], thus making it possible to address transferability of the RCT results to real-life. This publication report results on QoL, whereas results on costs will be reported in a subsequent article.

## Methods

### Treatment

Balloon kyphoplasty is a minimally invasive procedure of vertebral augmentation for the treatment of painful, acute VCF. Through a small incision in the patient's back, the surgeon creates a small pathway to the fractured bone with a hand drill. Through these channels, inflatable bone tamps can then be inserted into the medullary space and filled with a radiopaque contrast medium for visualization. The balloon is carefully inflated in order to restore the height and kyphotic angle of the collapsed vertebra; and thereafter; it is deflated and removed. The cavity created is filled in with cement at low pressure (typically polymethylmethacrylate) in order to support the surrounding bone, prevent further collapse and hold the restored vertebra in place. The procedure is done on both sides of the vertebral body and can be controlled by a digital meter for compression. Thanks to shape restoration and stabilization of the vertebral body an immediate pain relief can occur [6].

### Study design

Information on patients was collected during a multicenter, prospective, controlled, non-randomized study. From 2005 to 2008, 124 osteoporotic patients with VCF were treated in eight different study hospital centres and clinics in Germany with NSM or BKP. They were then followed over 12 months after intervention. Inclusion criteria were the following: Male or female aged 50 years or older with at least one painful thoracic or lumbar (T5 to L5) VCF due to osteopenia from primary or secondary osteoporosis. Fractures should be acute, not older than three months; and patients must have suffered from acute pre-treatment pain with VAS (Visual Analogue Scale) score no smaller than 5 on a scale of 10. Furthermore patients must have been available for follow-up interviews. Patients had to give their informed consent prior to collection of data. Exclusion criteria were any previous vertebroplasty or balloon kyphoplasty, pre-existing and disabling chronic back pain of different origin, or inability to walk or stand before the fracture occurred (walking aids allowed). Patients must not have dementia and/or inability to give informed consent, and unwillingness or inability to participate to the follow-up phone interviews.

### Health Outcomes

*Health-Related Quality of Life *(HR-QoL) is an important health outcome of spinal treatments, as relief of pain symptoms is the first aim of those treatments. Two sets of instruments were used to collect HR-QoL data: the EQ-5D as a generic health outcome instrument and the RMDQ as a specific-back pain related instrument.

The *EQ-5D *consists of five domains: mobility, self-care, usual activities, pain/discomfort and anxiety/depression. The respondent can choose between three levels corresponding to (1) no, (2) some or (3) extreme problems. The result is a health profile giving information on the health state of the patient in each of these 5 domains. This profile is transformed thanks to an algorithm to obtain a single score of health-related quality of life (also called utility level) ranging from 0 for the worst health state to 1 for perfect full health. Two different algorithms were used in this study to get those scores: the German TTO (Time Trade Off)-based algorithm [10] and the European VAS-based algorithm [11]. To assess relevant changes in HR-QoL measured by EQ-5D, the Minimally Clinically Important Difference (MCID) for EQ-5D in degenerative spinal conditions has been assessed at 0.08 points [7,12,13]. In addition, the EQ-5D includes the visual analogue scale (VAS) to capture the patients subjective HR-QoL perception on a scale ranging from 0 (worse health state) to 100 (perfect health) [10].

Finally, the RMDQ was used as a related-back-pain quality of life instrument with 24 questions. Affirmative answer to each question of the RMDQ is scored one, making higher scores associated with greater disability (with 0 for no disability to 24 for highest disability) [14-16]. The Minimally Clinically Important Difference (MCID) for RMDQ has been estimated to be 2 to 3 points for general degenerative spinal conditions [15].

### Data collection and Statistical methods

At baseline patient data was evaluated by questionnaires. For follow-up data patients were contacted by phone after 14 days and then every month up to 12 months. In case of incomplete or missing follow-up data, the last observation carried forward method (LOCF) was used [17]. For descriptive analysis and statistics Microsoft Excel and SPSS 16.0 was used. Difference in parameters between study groups was tested thanks to Mann-Whitney-U tests and deemed significant for p-values smaller than 5%.

## Results

### Socio-demographic and clinical patient's characteristics

Data from 124 patients was collected during the recruitment. 23 patients were eliminated due to missing contact or other reasons in the first three months of the study. 13 patients were lost during follow up ("drop-outs"): 4 patients died, whereas the remaining 9 declined or were not available at the FU visits. An analysis of the drop-outs showed no significant differences regarding age, EQ-5D baseline value and RMDQ. In total 88 patients finished the study („full-period"), but six patients had missing data for the EQ-5D. Therefore the final analysis was made for 82 patients: 46 were BKP-patients and 36 NSM-patients. Patients were aged from 52 to 85 years old, with a mean age of 71.9 years (BKP: 71.7 years; NSM: 72.0 years). 91.5% (BKP: 91.3%; NSM: 91.7%) of the patients were female. There were no statistically significant differences regarding age or gender between the study groups (p = 0.885/p = 0.954). Furthermore pain before surgery was evaluated. There were no significant differences between the groups (p = 0.217). However, length of hospital stay during follow-up was significantly longer in the NSM-group in comparison to the BKP-group (10.5 days vs. 17.9 days, p = 0.006)

### EQ-5D dimensions

**Figure 1:**
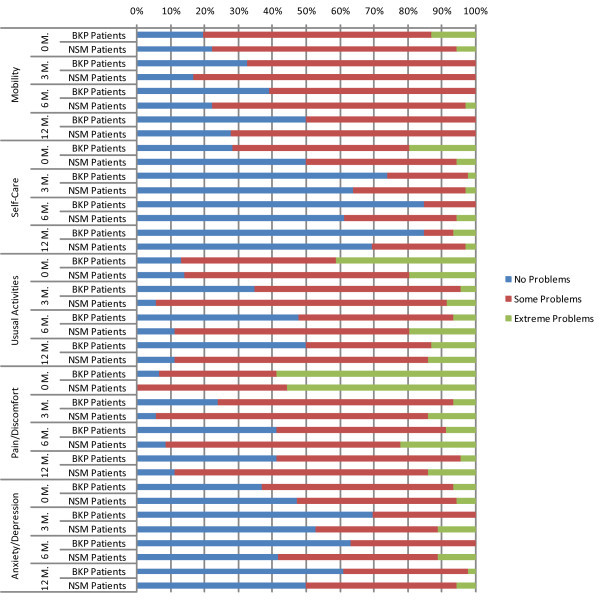
**Distribution of EQ-5D dimensions for BKP versus NSM patients**.

At *baseline*, in all EQ-5D dimensions except 'self-care' (p = 0.019) there were no significant differences between the study groups. Figure 1 presents the distribution within the EQ-5D dimensions for both study groups from baseline to 12 months after treatment. The results showed a trend in improvement in all domains of the EQ-5D during the follow-up, with more patients with no problem in all domains in the BKP group compared to the NSM one. At *12 months *more BKP patients had no problem in all domains than NSM patients. This improvement was statistically significantly higher for BKP patients then NSM ones, demonstrating thus a sustain improvement in mobility (p = 0.043), daily activities (p = 0.003), and pain (p = 0.002) still at 1 year after treatment.

### EQ-5D Visual Analogue Scale (VAS)

**Figure 2:**
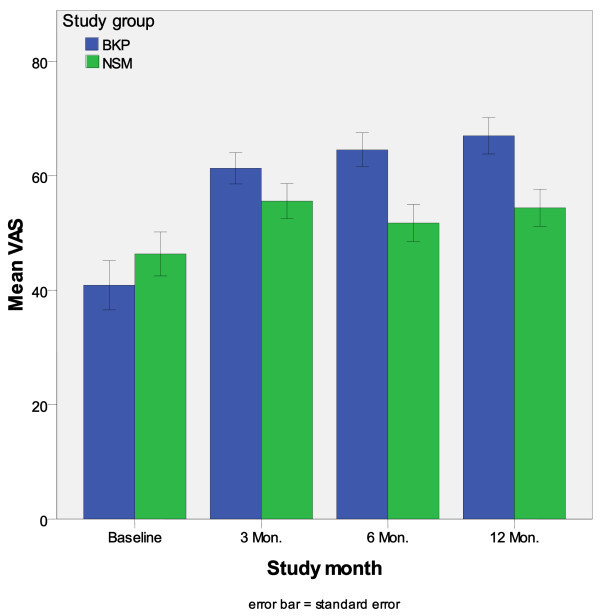
**EQ-5D Visual Analogue Scale (VAS) according to study groups**.

The EQ-5D VAS measures the subjective perception of the patients of their general own health state. There was no significant difference at *baseline *(p = 0.279) and 3 months (p = 0.252) in VAS scores between both treatment groups. At 6 and 12 months post-intervention BKP-patients had a significant higher improvement of their perceived health state compared to NSM patients (with respectively, p-value = 0.002 and 0.009). The mean EQ-5D VAS improved within the treatment and the control groups with respectively BKP (VAS: mean = 40.9; SEM (standard error of the mean) = 4.32 at baseline and VAS: mean = 67.0; SEM = 3.18 at 12 months) and NSM (VAS: mean = 46.3; SEM = 3.84 at baseline and VAS: mean = 54.4; SEM = 3.25) (see Figure 2).

### EQ-5D TTO (Germany) and EQ-5D VAS (Europe)

In terms of health-related quality-of-life, there were no statistically significant differences between study groups at baseline in the German and European EQ-5D scores (see tables 1 and 2).

**Table 1:** Mean EQ-5D TTO (Germany) for BKP and NSM, and differences between and within groups

	BKP group		NSM group		BKP vs NSM
**EQ-5D TTO German score**	**HR-QoL**	**Difference from baseline**	**HR-QoL**	**Difference from baseline**	**p-value of difference**

Baseline	0.370		0.435		0.206

3 months	0.774	0.404 †	0.651	0.217 †	0.008*

6 months	0.800	0.430 †	0.596	0.161 †	0.000*

12 months	0.812	0.442 †	0.686	0.251 †	0.008*

An asterisk * means that difference between treatment groups are statistically significant. A † means statistically significant difference from baseline.

**Table 2:** Mean EQ-5D VAS (Europe) for BKP and NSM, and differences between and within groups

	BKP group		NSM group		BKP vs NSM
**EQ-5D VAS European score**	**HR-QoL**	**Difference from baseline**	**HR-QoL**	**Difference from baseline**	**p-value of difference**

Baseline	0.395		0.449		0.132

3 months	0.689	0.294 †	0.570	0.121 †	0.010*

6 months	0.717	0.322 †	0.528	0.079 †	0.000*

12 months	0.723	0.328 †	0.597	0.148 †	0.004*

An asterisk * means that difference between treatment groups are statistically significant. A † means statistically significant difference from baseline.

Even though improvements from baseline in QoL were shown to be clinically and statistically significant in both groups, at all visits EQ-5D scores were statistically significantly better for BKP patients than NSM ones. In addition the improvement from baseline in the BKP group was higher at all time points than in the control group. From baseline to 1 year, the quality of life of BKP patients more than doubled from 0.37 to 0.81, whereas the NSM patients get a smaller increase from 0.43 to 0.68, with the German TTO algorithm. Those trends are also confirmed with the VAS-European valuation algorithm.

### Robert Morris Disability Questionnaire (RMDQ)

Back function was measured by the RMDQ. BKP patients clinically significantly improved their health status during the follow-up period, as measured by RMDQ from 15.18 at baseline to 8.93 at 12 months. On the contrary, NSM patients had no clinically relevant improvement in the specific-back function QoL instrument, as the RMDQ changed from 14.37 at baseline to 13.66 at 12 months, a variation of 0.71 points smaller than 2 to 3 points MCID.

As shown in table 3, there were no significant differences between the study groups at baseline. RMDQ scores were significantly better for BKP-patients at 3, 6 and 12 months within groups as well as in comparison to NSM.

**Table 3:** Mean RMDQ for BKP and NSM: differences between and within groups

	BKP group		NSM group		BKP vs NSM
**RMDQ score**	**RMDQ**	**Decrease from baseline (p-value Wilcoxon-test)**	**RMDQ**	**Decrease from baseline (p-value Wilcoxon-test)**	**p-value of difference**

Baseline	15.18		14.37		p = 0.318

3 months	10.27	4.91 (p = 0.000*)	14.40	*-0.03 *◊ (p = 0.392)	p = 0.004*

6 months	8.77	6.41 (p = 0.000*)	14.00	0.37 (p = 0.391)	p = 0.000*

12 months	8.93	6.25 (p = 0.000*)	13.66	0.71 (p = 0.370)	p = 0.001*

An asterisk * means that difference between treatment groups or time points are statistically significant. A † means statistically significant difference from baseline. A ◊ means, the RMDQ worsens from baseline.

A correlation analysis revealed highly significant correlations between RMDQ and EQ-5D values. This result suggests that both quality of life questionnaires reflect the differences in health for patients with osteoporotic VCF accordingly.

## Discussion

This study aimed at analyzing the discrepancy in health-related quality of life in VCF treated with BKP in comparison to the standard NSM treatment. Data was collected from a real-life prospective non-randomized controlled multicentre study in German healthcare setting. HR-QoL was assessed thanks to two main categories of instruments. Firstly, the EQ-5D generic health instruments with valuation from the TTO-German and VAS-European algorithm [10,11]. Secondly, the RMDQ, a disease-specific instrument was used to assess related-back function in those patients [14,15]. The analysis was performed on 82 patients (BKP: 46; NSM: 36), after considering drop-outs.

Based on those validated instruments, this study demonstrates a significant higher improvement in quality of life for BKP patients than for NSM ones. As expected improvements pre- and post-intervention were seen in both groups, however improvement was clinically more relevant from baseline to 12 months for BKP patients. In addition, back-related functional status as measured by the RMDQ significantly improved in the BKP group, whereas reached a plateau in the NSM group; therefore making BKP the preferred alternative treatment to cope with VCF-associated morbidity.

These results confirmed those of the IDE-FDA FREE trial, a randomized controlled study comparing BKP with NSM in VCF patients. In the German study results, patients had a higher quality of life at baseline than those in FREE. Despite this difference at inclusion, the improvement in EQ-5D scores were comparable in both studies, with +0.44 in the German study (from 0.37 at baseline to 0.81 at 12 months) and +0.45 in the FREE RCT (from 0.16 to 0.62^I^) for the BKP patients, and greater than for the NSM ones. Therefore, efficacy of BKP measured from RCT and its effectiveness in real-life settings appeared to be similar, validating BKP intervention. In addition, scores for disease-specific QoL instrument RMDQ were better at inclusion in this study compared to the RCT one. Improvement in RMDQ for BKP patients was also clinically and statistically significant in both studies, with a decrease in RMDQ of 6.25 in German hospital setting (from 15.2 at baseline to 8.9 at 12 months) and of 8.25 in the RCT (from 16.9 to 8.66^II^). However, difference in RMDQ improvement (from baseline to 1 year) between the BKP and the NSM groups was higher in this study, as NSM patients didn't improve functional status after NSM treatment. This difference could be due to both the small sample size of this German study without randomization and to the existence of a clear, binding NSM protocol in the RCT, according to centres practice, whereas in real-life NSM treatments are less standardized. Another limitation of this study could have been in the recruiting bias as patients were not randomized into the two treatment groups. However, patients were selected with the same inclusion criteria, and at baseline, no significant differences in QoL or demographic characteristics were found between groups. Furthermore despite a limited but statistically fair number of patients, patients were treated at several different clinics making bias less likely, which was reflected into highly significant differences in QoL results between study groups.

Given that this study has also registered medical resources consumption, and according to better quality of life and shorter hospital stays demonstrated for BKP over NSM, a cost-consequences analysis would be an asset for future research. In addition a longer period of follow-up of these patients could also be taken into account in a life-time perspective analysis of benefits and costs of BKP over NSM.

## Conclusions

This prospective controlled non-randomized study in German hospital setting demonstrates that effectiveness of BKP over time and compared to NSM is similar to its efficacy proven in RCT. The relative higher improvement in QoL outcomes and symptom's relief of BKP over NSM in patients with acute osteoporotic vertebral fractures proven in a randomized clinical trial seems to be captured in real-life. This QoL improvement in BKP patients appeared to be due to restoration of back function, as those patients had a statistically and clinically significant improvement in the back function-related instrument (RMDQ), which was not the case for NSM patients.

## Endnotes

^a^From FREE trial results; results not published; available upon request to Medtronic International.

^b^From FREE trial results; results not published; available upon request to Medtronic International.

## Competing interests

The data collection, analysis as well as the preparation of the manuscript was financed by an unrestricted research grant by Medtronic.

## Authors' contributions

DEK and WG developed the study design conjointly. DEK was responsible for the data acquisition and data analysis and was editing the manuscript.

WG reviewed the manuscript and formatted it.

JW/MK/SK helped to perform the statistical data analysis.
